# Host gene expression profiles in ferrets infected with genetically distinct henipavirus strains

**DOI:** 10.1371/journal.pntd.0006343

**Published:** 2018-03-14

**Authors:** Alberto J. Leon, Viktoriya Borisevich, Nahal Boroumand, Robert Seymour, Rebecca Nusbaum, Olivier Escaffre, Luoling Xu, David J. Kelvin, Barry Rockx

**Affiliations:** 1 Division of Experimental Therapeutics, Toronto General Research Institute, University Health Network, Toronto, Ontario, Canada; 2 Department of Pathology, University of Texas Medical Branch, Galveston, TX, United States of America; 3 Microbiology & Immunology, University of Texas Medical Branch, Galveston, TX, United States of America; 4 Institute of Human Infections and Immunity, University of Texas Medical Branch, Galveston, TX, United States of America; 5 Department of Microbiology and Immunology, Dalhousie University, Halifax, Canada; 6 International Institute of Infection and Immunity, Shantou University Medical College, Shantou, PRC; 7 Department of Viroscience, Erasmus University Medical Center, Rotterdam, The Netherlands; School of Veterinary Medicine University of California Davis, UNITED STATES

## Abstract

Henipavirus infection causes severe respiratory and neurological disease in humans that can be fatal. To characterize the pathogenic mechanisms of henipavirus infection *in vivo*, we performed experimental infections in ferrets followed by genome-wide gene expression analysis of lung and brain tissues. The Hendra, Nipah-Bangladesh, and Nipah-Malaysia strains caused severe respiratory and neurological disease with animals succumbing around 7 days post infection. Despite the presence of abundant viral shedding, animal-to-animal transmission did not occur. The host gene expression profiles of the lung tissue showed early activation of interferon responses and subsequent expression of inflammation-related genes that coincided with the clinical deterioration. Additionally, the lung tissue showed unchanged levels of lymphocyte markers and progressive downregulation of cell cycle genes and extracellular matrix components. Infection in the brain resulted in a limited breadth of the host responses, which is in accordance with the immunoprivileged status of this organ. Finally, we propose a model of the pathogenic mechanisms of henipavirus infection that integrates multiple components of the host responses.

## Author summary

Henipavirus infection can cause severe and often lethal respiratory and neurological disease in humans. The mechanisms by which these viruses cause disease are unknown. To characterize the mechanisms of henipavirus disease *in vivo*, we performed experimental infections in ferrets followed by characterization of the host response in lung and brain tissues. Characterization of the host response showed early activation of antiviral responses in the lung, in the absence of lymphocyte activation. Infection in the brain resulted in a limited breadth of the host responses, in accordance with the immunoprivileged status of this organ. Overall, infection with distinct henipavirus strains differed in magnitude of the host responses rather than activation of different pathways. Finally, we propose a model of the pathogenic mechanisms of henipavirus infection that integrates multiple components of the host responses.

## Introduction

Hendra (HeV) and Nipah (NiV) viruses (genus *Henipavirus*, family *Paramyxoviridae*) are zoonotic pathogens that can cause severe acute respiratory distress and neurological disease in humans [1]. HeV was initially identified during an outbreak of acute respiratory disease in horses in Australia in 1994. To date, 7 human cases of HeV infection have been identified, all associated with direct contact with infected horses and with a case fatality rate (CFR) of 57% [2]. NiV was first identified as the cause of an outbreak of acute respiratory and neurological disease in pigs in 1998–99 that led to infections in humans in Malaysia (283 cases) and Singapore (11 cases) with a CFR of 38% [3]. Subsequent outbreaks of NiV have occurred almost yearly in humans in Bangladesh and India with increased CFR (75%) and higher prevalence of respiratory disease (from 40% to 75%) [2, 4]. In addition, human-to-human transmission has only been observed during outbreaks of NiV in Bangladesh. The NiV strains isolated during outbreaks in Malaysia and Bangladesh are genetically distinct and have been designated as the Malaysia strain (NiV-M) and Bangladesh strain (NiV-B), respectively [5, 6].

In hamsters and other animal models, infection with HeV is generally more severe than infection with NiV-M and NiV-B [7, 8]. These possible differences in virulence between HeV and NiV-M have also been observed in the recently developed African green monkey model [9]. Interestingly, in that same model, a recent study showed that NiV-B is more pathogenic, similar to what is seen during outbreaks of human disease [10]. The differences in the pathogenic mechanisms of these strains, which ultimately drive the disparity of clinical outcomes and transmission profiles, remain largely unknown.

Ferrets are commonly used as experimental models of infection for a variety of respiratory viruses due to their susceptibility to these viruses and the close resemblance of the pathological features to those found in human infections [11, 12], including the development of severe respiratory and neurological disease during henipavirus infection [13–15]. Despite the limitations in ferret-specific reagents, the use of ferrets as experimental models of infection has gained interest since the publication of the ferret transcriptome and draft genome [16–18], which opened the doors to performing whole-genome gene expression analysis in this animal model.

In this study, we have performed a systematic characterization of HeV, NiV-B and NiV-M infection in a well-established lethal challenge model in ferrets and used next-generation mRNA sequencing to characterize the evolution of the host responses in key target organs, the lung and the brain. This analysis provides an integrated view of the functional components of the host responses that participate in the fight against the virus.

## Results

### Effect of infectious dose on experimental Henipavirus infection in ferrets

**Fig 1:**
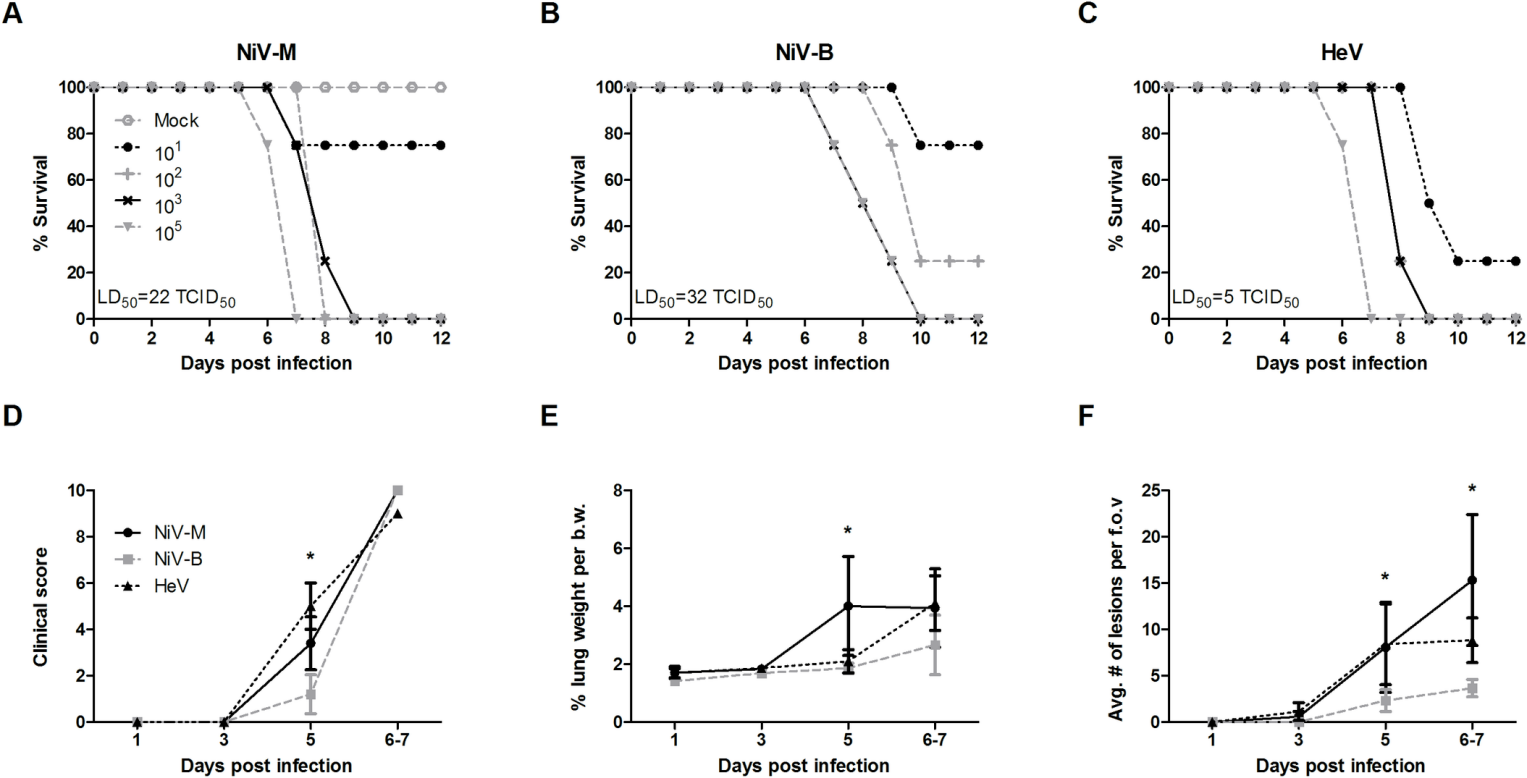
Differences in minimal infectious dose of HeV, NiV-M and NiV-B in ferrets. A first study using different infective doses of the three viruses was performed to establish the LD_50_ of each virus in ferrets: A-C) Survival rates are shown for 6-week old ferrets (n = 5 per group) that were infected with different doses (10^1^, 10^2^, 10^3^ and 10^5^ TCID_50_) of NiV-M, NiV-B and HeV, respectively. D-F) A comparative study using a dose of 5,000 TCID_50_ for each of the three viruses was performed in ferrets: D) cumulative clinical scores, calculated as described in the methods section; E) % lung weight per body weight; F) average number of histological lesions in the lung tissue per field of view. Tissues were harvested from 6-week old ferrets infected with NiV-B (grey), NiV-M (black solid) and HeV (black dashed) on various days post infection as described in Materials and Methods. Samples from 5 animals per group were analyzed at each time point. * p<0.01, two-way ANOVA, Bonferroni’s multiple comparison test. Error bars represent standard deviations.

In order to characterize infection of NiV-M, NiV-B and HeV in the ferret model and to determine the optimal challenge dose for subsequent studies, groups of 4 animals were challenged with 10-fold dilutions (10, 10^2^, 10^3^, or 10^5^ tissue culture infectious dose 50%, or TCID_50_) of these three henipavirus strains via the intranasal (IN) route. Clinical signs were first noticeable on day 5 post infection (pi), and included development of fever, labored breathing, mild paralysis, generalized tremors and subcutaneous edema of the head and neck, followed by rapidly progressing clinical signs on days 6 and 7 pi. This coincided with a small increase in body temperature of HeV and NiV-B infected animals on day 5 pi (S1 Fig). Moribund animals displayed clinical signs that included lack of grooming, hunched posture, ataxia, severe depression, labored breathing, occasional subcutaneous edema of the neck and head, vomiting; and neurological signs such as continuous licking, tremors, imbalance, myoclonus, head tilt, hind limb paralysis and seizures. The majority of animals succumbed of infection by day 6–8 pi with a lethal dose 50% (LD_50_) for HeV, NiV-B and NiV-M of 5, 32 and 22 TCID_50_, respectively (Fig 1A–1C). Several ferrets receiving low infective doses (10 or 10^2^ TCID_50_) displayed no clinical signs throughout the period of observation and did not seroconvert, suggesting that the LD_50_ is also the minimal infectious dose at which 50% is infected.

### Pathological features of experimental henipavirus infection in ferrets

For characterization of infections with three genetically distinct henipavirus strains at virological, pathological and gene expression levels, a serial sacrifice study was performed using groups of 5 ferrets that were challenged with 5,000 TCID_50_ of each virus via the IN route.

On day 5 pi, the animals showed the first signs of disease, and although at this time-point ferrets infected with HeV and NiV-M presented higher cumulative clinical scores, as compared to those with NiV-B infection, they all reached a similar level of severity when animals were moribund by day 6–7 pi (Fig 1D).

In terms of gross pathologic changes, NiV-M and HeV-infected ferrets displayed small pinpoint hemorrhages in the lung tissue as early as day 3 pi, and large areas of hemorrhagic lesions by day 5 pi. On the other hand, NiV-B infection caused small pinpoint hemorrhages by day 5 pi that progressed into large hemorrhagic lesions in the moribund animals by day 6–7 pi. Lung wet weights were previously shown to be a good indicator of inflammation and edema [8]. In NiV-M infected animals, lung weight was significantly increased on day 5 pi compared to NiV-B and HeV infected animals (Fig 1E), whereas in moribund animals, lung weights were increased in all infected ferrets regardless of which strain was used. Gross pathologic changes in other organs included hemorrhages in the spleen and kidney as well as congestion of vessels in the brain in NiV-M and HeV-infected animals. Enlarged cervical and mesenteric lymph nodes in NiV-M infected ferrets were also noted. In NiV-B infected ferrets, no gross pathologic changes were observed in organs other than the lung.

Immunohistochemical analysis revealed that, by day 3 pi, NiV-M and HeV infection resulted in small foci of viral antigen in several of the lungs (Fig 1F). These foci increased in number and size over time and, by day 5 pi, they were significantly more numerous and larger in size in the animals infected with NiV-M and HeV as compared to NiV-B. Though no histologic changes were noted in the brain during the entire time course, foci of viral antigen were present (S2 Fig).

### Virus replication and tissue tropism of HeV, NiV-B, and NiV-M in ferrets

**Fig 2:**
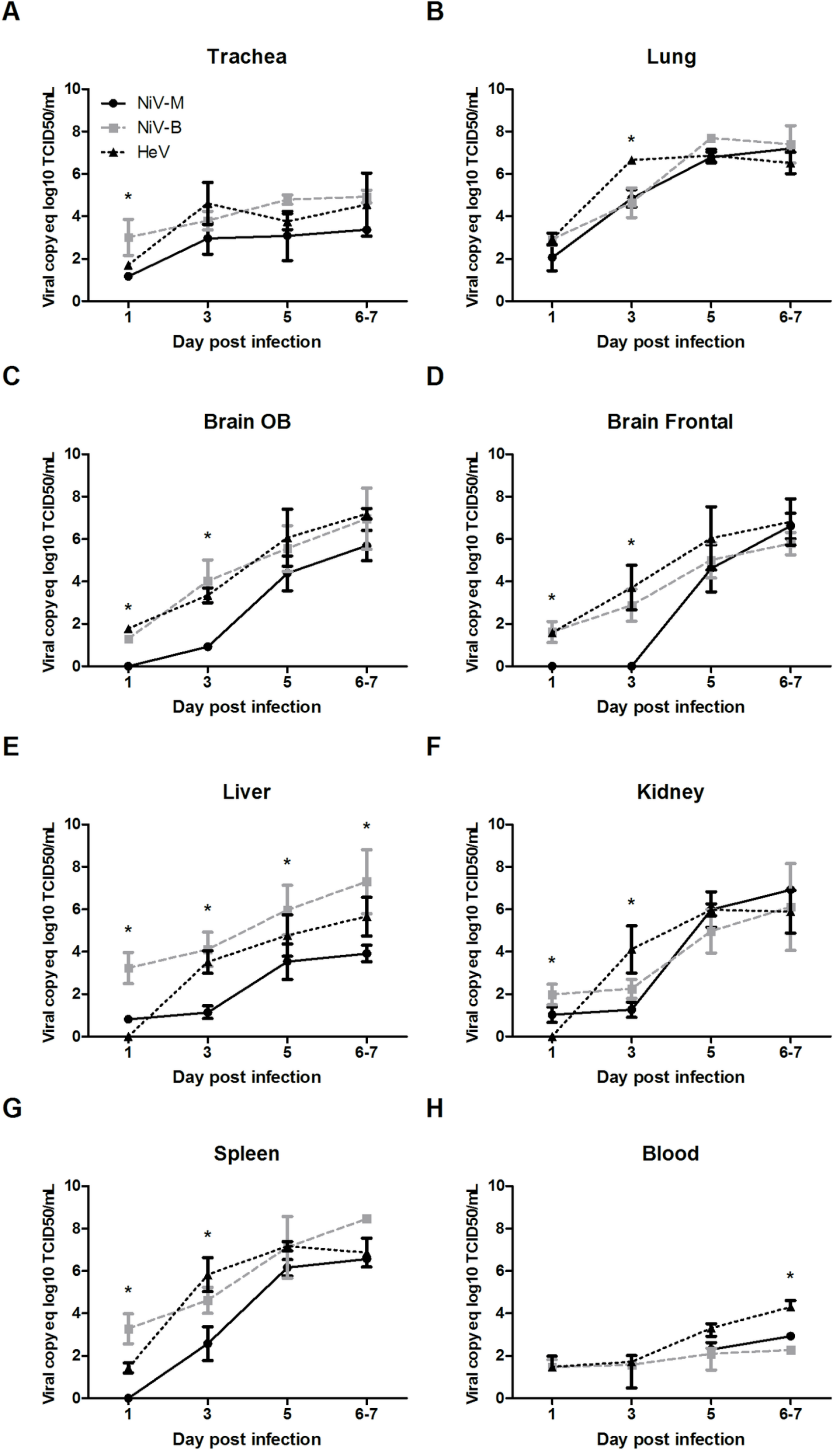
Viral spread across body compartments in henipavirus infected ferrets. A comparative study using a dose of 5,000 TCID_50_ for each of the three viruses was performed in ferrets: viral growth in the ferret tissue, as measured by quantitative RT-PCR in the trachea (A), lung (B), olfactory bulb (C), frontal brain (D), liver (E), kidney (F), spleen (G), and blood(H); tissues were harvested from 6-week old ferrets infected with NiV-B (grey), NiV-M (black solid) and HeV (black dashed) on various days post infection as described in Materials and Methods. Samples from 5 animals per group were analyzed at each time point. * p<0.01, two-way ANOVA, Bonferroni’s multiple comparison test. Error bars represent standard deviations.

Levels of viral RNA were determined in all tissues collected at necropsy by quantitative RT-PCR. Viral RNA was detected in all collected tissues (trachea, lung, olfactory bulb, frontal brain, cerebellum, liver, spleen and kidney) and in the blood, starting as early as day 1 pi (Fig 2A–2H). Overall, all three henipavirus strains showed a progressive increase in levels of viral RNA over time in all organs with the highest levels of replication detected at the time when animals were moribund.

When compared to NiV-M, significant higher levels of NiV-B RNA were detected in the trachea, OB, FB, and spleen at an early stage of infection, and until 3 dpi in the latter. This was also true in the liver throughout the infection (Fig 2A–2H). Likewise, when compared to both NiV strains, significant higher levels of HeV RNA were only transiently observed in lung and kidney at 3dpi, as well as in blood when animals were moribund at 6-7dpi (Fig 2A–2H). When solely compared to NiV-M, significant higher levels of HeV RNA were noted in the OB, FB, and spleen from 1 to 3 dpi.

### Absence of animal-to-animal transmission despite abundant viral shedding

To characterize the viral shedding profile of infected animals and to study potential animal-to-animal transmission, groups of 4 ferrets were infected IN with 5,000 TCID_50_ of HeV, NiV-B and NiV-M, and each of these groups was co-housed with 4 naïve ferrets. Levels of viral RNA were determined in nasal, oral and rectal swabs and urine by quantitative RT-PCR, and these samples were also tested for presence of infectious virus. All the infected ferrets were euthanized due to severe disease on day 5 and 6 pi (dpi). In the infected animals, low levels of viral RNA were detected in nasal washes for all viruses by 3 dpi (S3A Fig). Interestingly, on 5 dpi, shedding of NiV-M and HeV were significantly higher in nasal washes compared to NiV-B. Once animals were moribund, no significant differences in viral shedding in nasal washes were observed between the 3 virus strains. Shedding of HeV in oral swabs was significantly higher compared to NiV-B on 5 dpi and NiV-M when the animals were moribund (S3B Fig). Very low levels of viral RNA were detected in rectal swabs at early time-points; however, once animals were moribund, HeV shedding was significantly higher compared to NiV-B (S3C Fig). Finally, shedding of all 3 viruses was also detected in urine, with significantly higher NiV-B shedding on 1 and 3 dpi compared to NiV-M (S3D Fig).

The naïve ferrets that were co-housed with the infected animals showed no evidence of weight-loss or clinical signs, and they were euthanized three weeks post initial exposure. Their nasal, oral, and rectal swabs remained negative for virus throughout the study, and no virus specific antibodies were detected by ELISA or neutralization assay (S1 Table), suggesting that asymptomatic infection did not occur following direct contact with infected animals. These results indicate that the observed virus shedding that takes place during henipavirus infection does not translate in efficient animal-to-animal transmission in this model.

### Host gene expression in the lung tissue of henipavirus infected ferrets

To characterize the host responses following infection with two genetically and phenotypically distinct henipaviruses, we performed whole-genome gene expression analysis on the lung and the brain tissues, which constitute viral targets of clinical relevance. HeV and NiV-B viruses were selected for transcriptome analysis since they represented the most and least virulent henipaviruses in our model, respectively.

**Fig 3:**
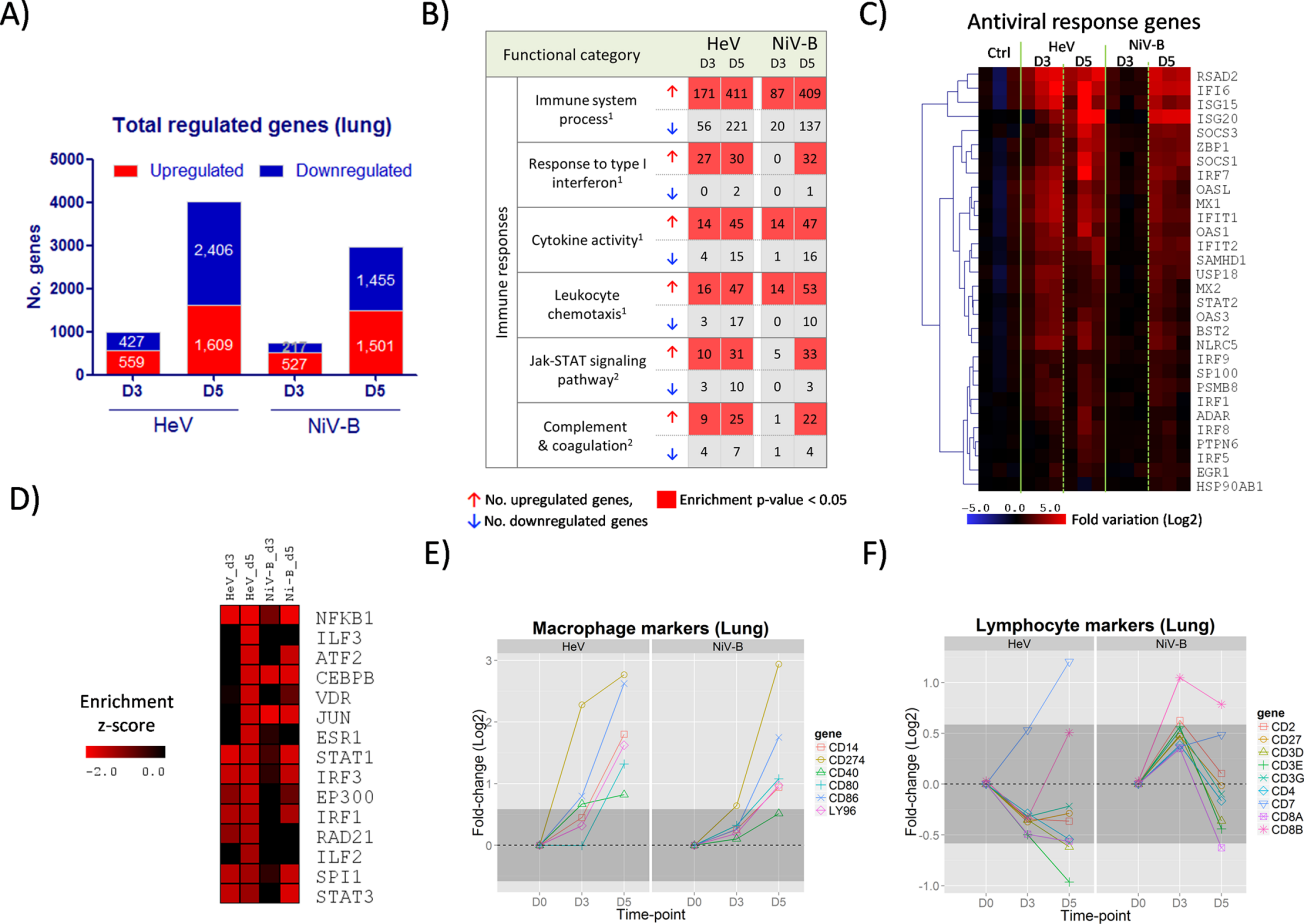
Gene expression profiles in the lung tissue of ferrets infected with HeV and NiV-B. Ferrets were infected with 5,000 TCID_50_ of HeV and NiV-B and euthanized at 3 and 5 d.p.i. and together with an uninfected control group (n = 3 per group). Transcriptomic analysis of the lung tissue was performed by RNA-seq. A) Number of significantly up- and down-regulated genes with respect to the control group (fold-variation>±1.5 and FDR<0.05) at 3 and 5 dpi. B) Functional gene classification of differentially expressed genes. For each time-point, up- and down-regulated genes were subjected to functional classification and a selected group of gene categories from ^1^Gene Ontology and ^2^KEGG Pathways that are related with the immune responses are shown. For each functional category, the number of up- and down-regulated genes and the level of significance of the enrichment (Fisher’s exact test p-value) are indicated. C) Heatmap showing the level of activation of genes belonging to the Response to Type I Interferon category, which encompasses antiviral response genes, during the course of HeV and NiV-B infection. D) Functional enrichment of up-regulated genes that belong to protein-protein interaction (PPI) hubs of transcription factors. E-F) Evolution in the mRNA expression levels of selected macrophage markers and lymphocyte markers, respectively, during HeV and NiV-B infection. These sets of markers were selected from the literature according to their association with these two cell populations, and their coordinated upregulation reflects an increase of the associated cell population in the tissue. Dark grey shaded areas indicate no variation with respect to the control group (<±1.5 fold-change).

The lungs of infected ferrets with HeV and NiV-B showed a progressive increase in the numbers of differentially expressed genes until 5 dpi, which was the last time-point analyzed (Fig 3A). Gene activation at the global level, and of the group of genes related to immune responses (Fig 3B), occurred later during NiV-B infection as compared to HeV, paralleling the later development of clinical signs observed in NiV-B infection. Specifically, HeV caused extensive activation of interferon stimulated genes (ISGs) (classified as Gene Ontology: Response to Type I Interferon) by 3 dpi that remained active by 5 dpi (27 and 30 upregulated ISGs, respectively); on the other hand, the activation of the antiviral responses during NiV-B occurred at a later time-point (0 upregulated ISGs by 3 dpi, and 32 by 5 dpi) (Fig 3B and 3C).The activation of other immune-related gene categories, including Cytokine Activity, Leukocyte Chemotaxis, Jak-STAT Signaling Pathway and Complement and Coagulation Cascades, also reached their highest degree of activation by 5 dpi with NiV-B causing a later start of these processes (Fig 3B). Also, enrichment of genes that belong to the protein-protein interaction (PPI) hubs of transcription factors, such as members of the STAT and IRF families, confirms the activation of genes related to immune responses in general and antiviral responses in particular (Fig 3D). Overall, these results indicate that the changes in the gene expression profiles in the lung tissue occurred in a time-dependent manner that parallels the replication kinetics of each virus and disease onset; NiV-B displayed a later activation of the immune responses as compared to HeV, but by 5 dpi, the two viruses display very similar profiles of gene activation both qualitatively and quantitatively.

The analysis of changes in the immune cell responses in the lung tissue was approached by assessing the level of gene expression of a set of selected cellular markers. As a group, macrophage markers (CD14, CD40, CD80, CD86, CD274 and LY96) showed marked upregulation at 5 dpi during both HeV and NiV-B infection (Fig 3E), indicating that progressive activation of the innate cellular responses takes place in this tissue. Interestingly, levels of lymphocyte markers (CD2, CD3D, CD3E, CD3G, CD4, CD7, CD8A, CD8B and CD27) remained mostly unchanged in the lung tissue during infection with both viruses (Fig 3F). Additionally, mRNA levels of CD3E and CD8A were also found to remain unchanged when analyzed by qPCR, whereas those from CXCL10, OAS1 and IFNγ showed a progressive increase, as expected (S4 Fig). These data of the evolution of cellular markers at the mRNA level suggest a deficit of activation of the local lymphocyte responses in the lung tissue.

**Fig 4:**
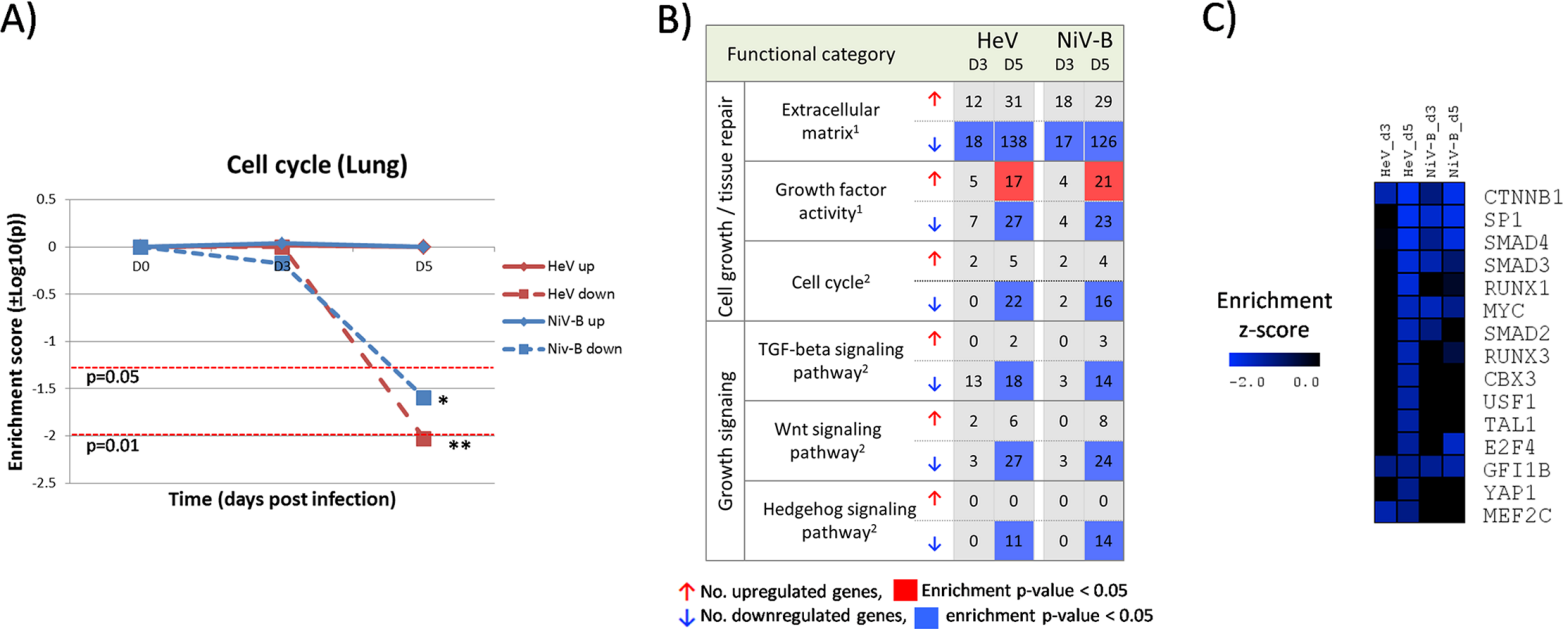
Decreased expression of genes related with cell cycle and growth signaling in the lung tissue during infection with HeV and NiV-B. A) Evolution of gene enrichment score (Fisher’s exact test p-value) of the Cell Cycle KEGG Pathway throughout the infection. B) Functional gene classification of differentially expressed genes in the lung tissue. Regulated genes were subjected to functional classification and selected ^1^Gene Ontology categories and ^2^KEGG Pathways that are related to the immune responses and cell growth signaling are shown. For each functional category, the number of up- and down-regulated genes and the level of significance of the enrichment (Fisher’s exact test p-value) are indicated. C) Functional enrichment of down-regulated genes that belong to protein-protein interaction (PPI) hubs of transcription factors.

Functional classification of the downregulated genes in infected lung tissues revealed a marked reduction in the expression levels of genes that participate in the cell cycle that fall below the basal levels of the uninfected controls. This phenomenon occurs progressively and reaches the strongest level of gene repression on 5 dpi for both HeV and NiV-B (Fig 4A). Genes of the Extracellular Matrix functional category, such as collagen proteins and other structural genes related to tissue repair, also display a marked downregulation during Henipavirus infection (Fig 4B). Moreover, gene members of the TGF-beta, Wnt/beta-catenin and Hedgehog signaling pathways, which are directly involved in the regulation of growth and cell cycle, were also downregulated. This scenario is further confirmed by enrichment of genes that belong to the protein-protein interaction hubs of transcription factors such as CTNNB1, SMAD3 and SMAD4 (Fig 4C), which are central components of the TGF-beta and Wnt/b-catenin signaling pathways. We also found that the expression landscape of genes with growth factor activity, some of which may be impacting the regulatory environment of the lung, is split towards up- and down regulation (Fig 4B).

### Host gene expression in the brain tissue during henipavirus infection

**Fig 5:**
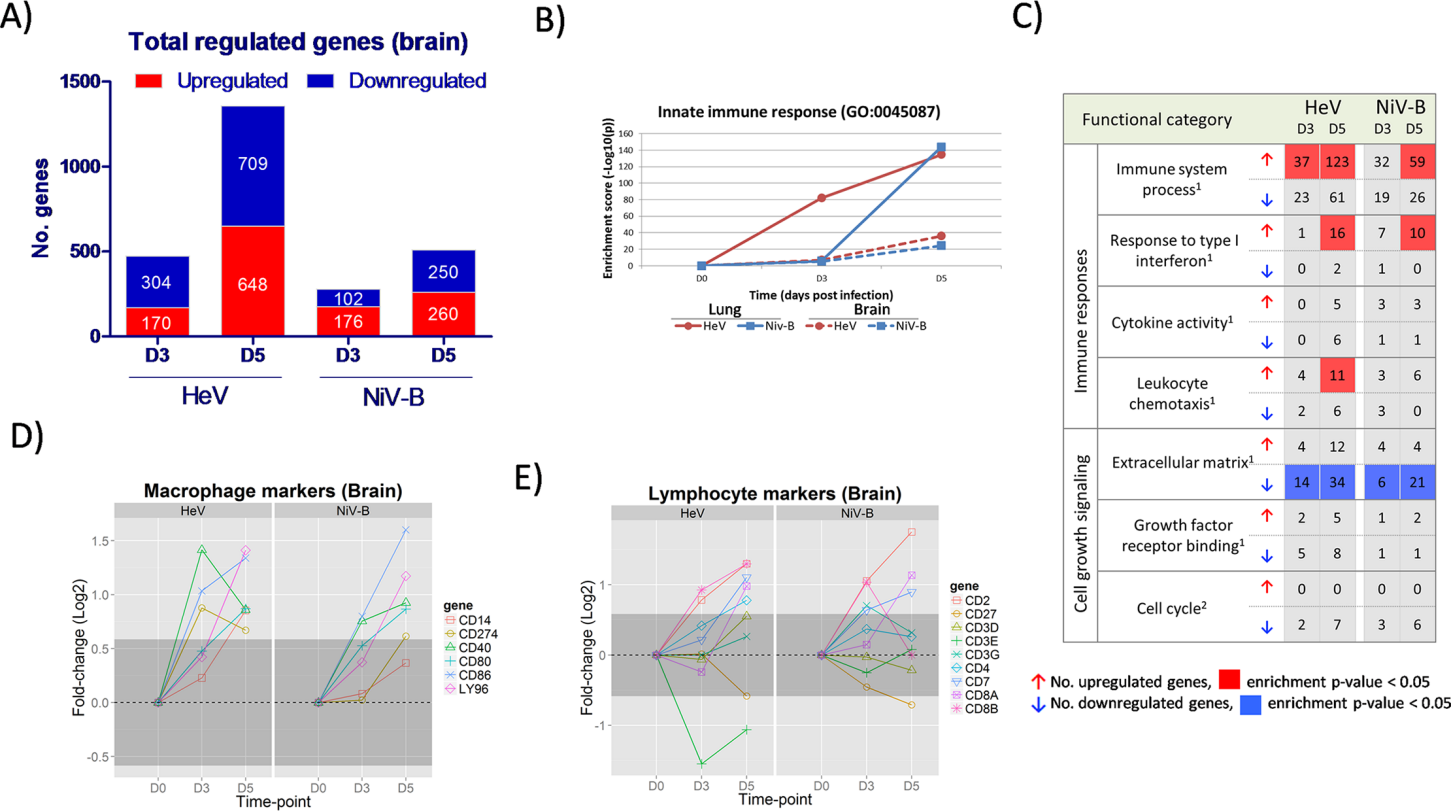
Gene expression profiles in the brain of ferrets infected with HeV and NiV-B. Ferrets were infected with 5,000 TCID_50_ of HeV and NiV-B and euthanized at 3 and 5 d.p.i. (n = 3 per group, except for HeV_D5 with n = 2) and together with an uninfected control group (n = 2). Transcriptomic analysis of frontal lobe tissue was performed by RNA-seq. A) Number of significantly up- and down-regulated genes with respect to the control group (fold-variation>±1.5 and FDR<0.05) at 3 and 5 d.p.i. B) Evolution of gene enrichment score (Fisher’s exact test p-value) of the Innate Immune Response category (Gene Ontology) throughout the infection. C) Functional gene classification of differentially expressed genes in the brain tissue. Regulated genes were subjected to functional classification and a selected ^1^Gene Ontology categories and ^2^KEGG Pathways that are related to the immune responses and cell growth signaling are shown. For each functional category, the number of up- and down-regulated genes and the level of significance of the enrichment (Fisher’s exact test p-value) are indicated. D-E) Evolution in the mRNA expression levels of selected macrophage markers and lymphocyte markers, respectively, during HeV and NiV-B infection in the brain tissue. Dark grey shaded areas indicate no variation with respect to the control group (<±1.5 fold-change).

The total numbers of differentially expressed genes in the brain frontal lobe increased progressively, but evolving faster and reaching higher levels by 5 dpi during HeV infection as compared to NiV-B (Fig 5A). Functional enrichment of regulated genes in during HeV and NiV-B shows the progressive activation of genes belonging to the Innate Immune Response and Immune System Process categories that occurs in parallel with the global gene activation (Fig 5B and 5C). Interestingly, we observed enrichment of downregulated genes that belong to the extracellular matrix category (Fig 5C), which can be related to homeostatic alterations in the brain tissue. In terms of cellular immune responses, expression of selected macrophage markers increased progressively during infection with both viruses (Fig 5D), and also, a number of lymphocyte markers were found to be upregulated in HeV at 5 dpi, whereas a more restricted activation of this group of genes was observed in NiV-B infection (Fig 5E).

## Discussion

HeV and NiV are emerging zoonotic viruses that can cause severe acute respiratory distress and acute encephalitis in humans. Outbreaks of infection with genetically distinct henipavirus strains in Malaysia (NiV-M), Bangladesh (NiV-B) and Australia (HeV) are associated with differences in clinical outcome and transmission patterns [2]. To better characterize their distinctive clinical features and understand their pathogenic mechanisms of disease, we performed experimental infections in a model of young ferrets, followed by analysis of their gene expression profiles in the lung and brain tissues. In these experiments, the three strains produced uniformly lethal infection and similar clinical signs that included severe respiratory and neurological disease. We also observed that the lungs, kidneys, liver and spleen were important targets of infection, suggesting that multiorgan failure was a likely cause of death, similar to that observed in the human cases that developed severe respiratory disease [19] and in other animal models of henipavirus infection [8, 9, 13, 20, 21]. Interestingly, despite the severe disease and viral shedding, no evidence of animal-to-animal transmission was observed. This is in agreement with previous studies showing a lack of transmission in the presence of viral shedding [22]. Based on the LD_50_ for each of the three viruses, HeV was most virulent, followed by NiV-M and where NiV-B required the highest dose to achieve 50% mortality, a scenario that is in accordance with the findings from previous reports [7–9]. The LD_50_ of NiV-M was lower compared to previous reports [15]. The use of younger animals in the current study may have had an effect on the susceptibility to these viruses resulting in a lower LD50 (for NiV-M) then previously reported. In addition, the immune system is still developing in young animals and as such may respond differently to outside stimuli. For instance, young ferrets are less susceptible to severe disease following influenza or SARS-CoV challenge compared to adults [23, 24]. This difference is believed to result from differences in host responses. However, in the current study, clinical signs, histopathological changes and other parameters are very similar to what has been reported in adult animals. Therefore, while this can only be confirmed by side-by-side comparison studies of young and adult animals, we believe that many of the results reported here, will be applicable regardless of age. Another variable that may affect the challenge efficacy is the volume used for the intranasal inoculation. The volume may affect the consistency to establish infection in a host, however the volumes used in other studies have not been reported. In order to increase the consistency of infection, we and others have used the same challenge dose of 5,000 TCID_50_, which results in a very robust challenge model [15, 25]. While the relative challenge dose may differ for each of the 3 henipavirus strains, given that they have different LD_50_, no correlation has been observed between dose, clinical outcome, histopathological changes and virus replication [15, 25].

### Early immune responses in the lung tissue during henipavirus infection

The general process of initiation and escalation of the host responses in the respiratory tract during henipavirus infection is comparable to that observed during infection with other respiratory viruses [26, 27]. After performing IN inoculation, HeV and NiV infect the epithelial cells of the upper respiratory tract, the trachea, bronchi and alveoli [8, 28]. Then, henipavirus infected epithelial cells produce type I interferon responses [29] that activate the antiviral responses, and cytokines that activate different components of the humoral and cellular innate immune responses at the tissue level [8, 28, 30–32]. As the respiratory infection progresses, the intensity of the local responses escalate beyond a biological threshold and triggers the activation of the endothelial cells, which, in turn, orchestrate and amplify the cytokine and chemokine responses leading to systemic immune activation, as shown for influenza virus [33].

The early activation of interferon responses is critical to control viral infections. For instance, it has been shown that ablation of the interferon signaling pathway in IFNA-R^-/-^ mice leads to the loss of the resistance to henipavirus infection that wild-type mice naturally possess [34]. During the early stages of henipavirus infection in ferrets, we observed robust expression of genes with antiviral activity in the lung tissue, including MX1, RSAD2, ISG15 and OAS1 (Fig 3C and S4 Fig), that was not sufficient to contain the spread of the virus.

The onset of inflammatory responses is mediated by the activation of the NF-κB signaling pathway, which is initially activated by the viral sensing mechanisms (RIG-I/MDA5 and TLR3) within the infected cells and later amplified and sustained by the inflammatory activity in the infected tissue [35]. Henipavirus infection results in increasing expression and broadening repertoires of mediators of inflammation, including cytokines, chemokines, CAMs, and also, increased expression levels of macrophage markers (CD14, CD40, CD80, CD86, CD274 and LY96) due to the influx of these cells into the infected lung tissue (Fig 3E). The development of tissue damage also coincided with the onset of clinical signs (Fig 2B–2D), and is possibly driven by the strong activation of NF-kb mediated responses that lead to high levels of macrophage and neutrophil infiltration in the lung, a scenario that is commonly found in respiratory infections of viral origin [8, 9, 21, 32, 36]. One caveat in this study is that tissues used for host gene expression analysis were not perfused to remove blood which could potentially “contaminate” host gene expression profiles. However, given that the levels of viremia were very low, this effect was not considered to be an important factor in characterizing the host response.

### Adaptive immune responses in the lung tissue during henipavirus infection

The initiation of the adaptive immune responses is a sequential process that includes antigen capture, processing and presentation, followed by lymphocyte selection and clonal expansion in the lymphoid tissues, and migration to the effector sites [37]. As part of this process, the influx of lymphocytes into the infected lung tissue is commonly observed during experimental infections *in vivo*; for instance, this process is detectable during influenza infection in mice by 4 dpi (increased levels of CD8+ and CD19+ cells) [38, 39], and in ferrets by 5 dpi (increased mRNA expression of lymphocyte markers) [17]. Strikingly, we found that the infection with HeV and NiV-B did not modify the expression levels of lymphocyte markers (CD2, CD3D, CD3E, CD3G, CD4, CD8A and CD27) in the lung tissue by 5 dpi (Fig 3F). These results point to a deficit or delay of lymphocyte activity in the lung; additional studies are required to profile the behavior of the different lymphocyte populations in the lung and their subsequent functional implications in the context of henipavirus infection.

The local regulatory environment may contribute to this possible deficit in the influx of lymphocytes into the lung. The observed reduction in levels of secreted activators of the Wnt/β-catenin pathway can decrease the local induction and maintenance of regulatory lymphocyte responses [40]. TGFβ exerts multiple activities that balance the immune responses, on one side it has immunosuppressive activities that normally prevent excessive activation of the immune responses; on the other hand, TGFβ signaling also exerts activating roles that are necessary to sustain the activation of regulatory and effector lymphocytes [41], and whose absence during henipavirus infection might contribute to dysregulate the local lymphocyte activity.

The hypothesis that henipavirus infection results in a deficit in the lymphocyte responses was previously postulated by Berhane *et al*. in the context of experimental NiV infection that caused lymphoid depletion in the lymph nodes and promoted susceptibility to bacterial co-infection [42]. The capacity of henipaviruses to spread to lymphoid tissues, including the infection of lymph nodes [42] and the infection of the spleen that was found in the present study (Fig 2J) and also by others [43], has been previously linked to a subsequent loss of immune responsiveness [43]. Nonetheless, it has not yet been established whether this capacity of henipaviruses to infect lymphoid organs is the result of non-specific systemic spread, or mediated by selective targeting of immune cells; if confirmed, the latter scenario would bear resemblance to that observed in measles infection, where the capacity of the virus to target immune cells [44] contributes to the hallmark immunosuppressive capability of measles virus [45, 46].

### Lack of activation of tissue repair mechanisms in the lung tissue during henipavirus infection

Under physiological conditions, a balanced production of the multiple components of the extracellular matrix is required to maintain the correct organization and functioning of the lung tissue [47]. When lung injury occurs, different processes such as collagen deposition and subsequent metallopeptidase-driven digestion are set in motion to preserve the structure of the tissue, and, together with cell growth, restore the structural integrity of the tissue and recover the basal levels of functionality [36]. This general process has been observed at the transcriptional level in the lung tissue during influenza infection in ferrets, where the activation of these mechanisms of tissue repair occurs along with the development of tissue inflammation and they remain active after the virus has been cleared to restore the basal conditions [17].

Strikingly, we found that infection with HeV and NiV-B results in progressive downregulation of genes related to the cell cycle, extracellular matrix components (including collagen subunits and other proteinaceous components of the extracellular matrix) and metallopeptidase genes in the lung tissue (Fig 4). Additionally, the signaling environment also points to a lack of activation of the effector tissue repair mechanisms, as indicated by the significant downregulation of genes belonging to the TGFβ, Wnt/β-catenin and hedgehog signaling pathways (Fig 4B) which are directly involved in the response to tissue damage through tissue repair and homeostasis [48, 49] (Fig 4B). To our knowledge, this regulatory and functional landscape has not been previously described in the context of an infectious process. Additional research is required to evaluate the impact that these changes at the transcriptional level exert at the physiological level, and to elucidate their involvement in the exacerbation of the respiratory disease during henipavirus infection.

### Host responses in the brain tissue during henipavirus infection

As part of the general process of brain infection by RNA viruses, infected neurons and other cell types, produce IFNs that activate the antiviral responses in an autocrine and paracrine manner, and secrete a restricted set of cytokines that includes IL6 and CX3CL1, which activate the neighboring astrocytes and microglia. In turn, these cells express MHC molecules and additional cytokines and chemokines, including IL1, IL6, IL12, TNFα, CCL2 (MCP1), CCL5 (RANTES) and CXCL10, that lead to the activation of the capillary endothelial cells; these cells express adhesion molecules, and together with the newly formed cytokine/chemokine gradient, lead to an infiltrate of different mononuclear cell populations, including NK, monocyte/macrophages and lymphocytes [50].

In our experimental model of henipavirus infection, the transcriptional landscape of the ferret brain is characterized by a progressive increase in the expression levels of ISGs and genes related with the innate immune responses that occurs in parallel with the development of neurological clinical signs. This is in accordance with previous observations in Syrian hamsters where IL-1β and TNF-α expression in the brain correlate with an increase in blood brain-barrier permeability and development of neuroinflammation [8]. We observed robust increase in macrophage markers (Fig 5D), however, the results of lymphocyte markers by RNA-seq and RT-PCR do not allow to reach conclusions regarding the influx of lymphocytes into the brain tissue. While lymphocytes have been implicated in playing a role in dissemination of henipaviruses in the host [51, 52]; additional experiments will be necessary to determine the exact profile of lymphocytes that migrate into the brain tissue and to establish their role in henipavirus pathogenesis. Other than the earlier activation of the host responses during HeV infection, as compared to NiV-B infection, no qualitative differences in the brain responses of the host were observed.

### Conclusions

**Fig 6:**
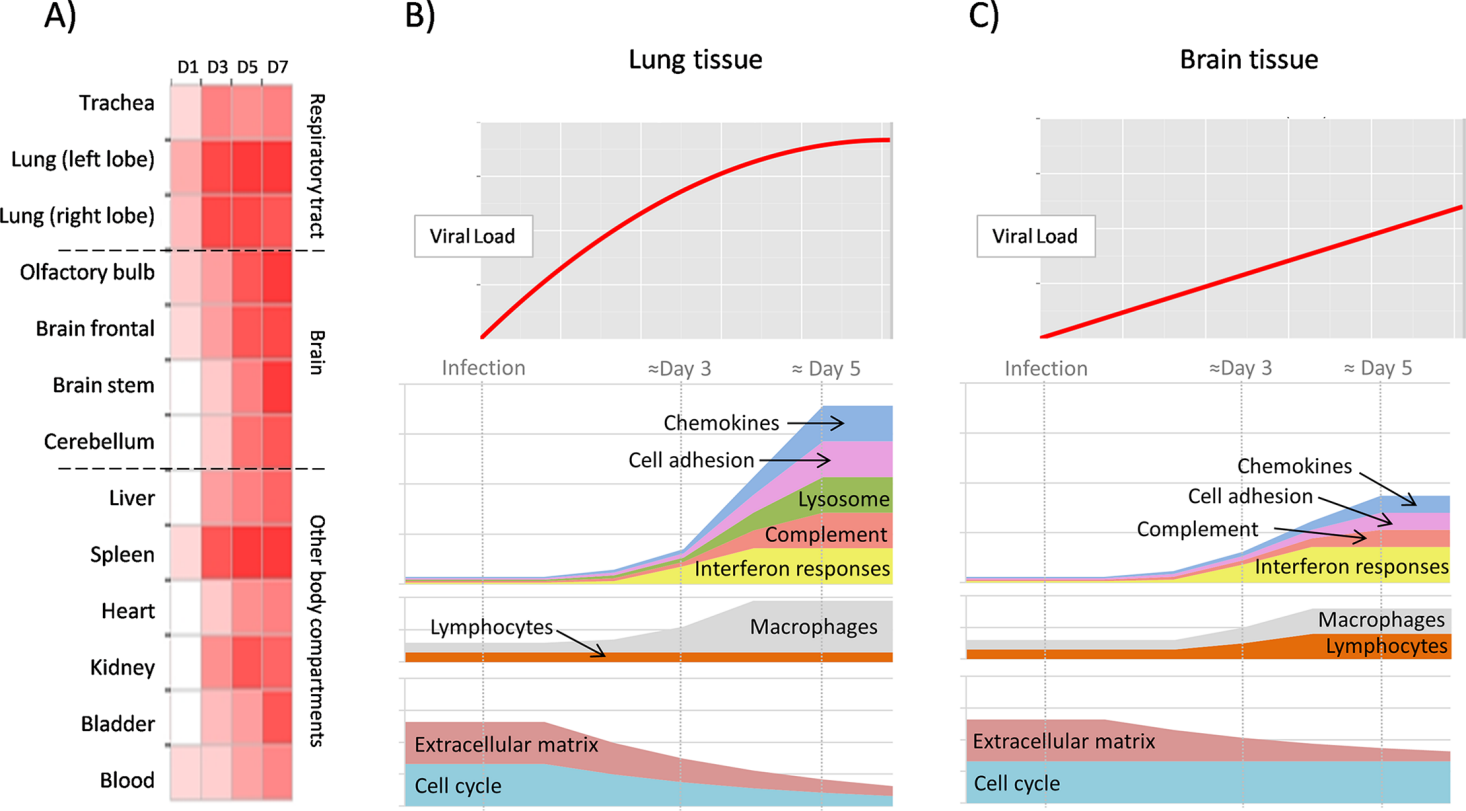
Sequential activation of host responses during Henipavirus infection. A) Upon infection of the upper and lower respiratory tract, the virus quickly spreads to a myriad of organs, including the central nervous system, liver, spleen, heart, kidney, bladder and blood (schematic representation of the data from HeV infection). B) In the lung tissue, the virus presents high growth rates and only after the inflammatory responses become fully active, at around day 4, the levels of virus stabilize but without significantly decreasing. Signaling and effector molecules of the innate immunity become expressed, innate immune cells migrate to the lung tissue, but, interestingly, gene expression data strongly suggest that lymphocytes are not migrating and expanding in the affected tissues. Additionally, the expression of genes related to the cell cycle, growth factors and growth factor signaling decline through the infectious process, possibly related to the degradation of the physiological functions of the lung tissue. C) In the brain tissue, the virus grows at a slower rate as compared to the lung tissue. The brain mounts a restricted immune response in accordance with the immunoprivileged status of this tissue that includes infiltration and/or local expansion of macrophages, and possibly of lymphocytes, but without leading to a noticeable alteration of the viral growth kinetics.

This study is the first comprehensive characterization of the host gene expression during henipavirus infection, by taking advantage of the recently published ferret genome and transcriptome and a well characterized ferret model for henipavirus infection. Overall, these data show that genetically and phenotypically distinct henipavirus strains differ in the kinetics of disease and host responses, rather than the magnitude. This is similar to observation with different SARS-CoV strains [24, 53]. Infection with henipaviruses, is associated with a strong host innate immune response, which contributes to severe respiratory stress and lethality. Fig 6 recapitulates the major findings in terms of viral spread across body compartments and host responses in the lung and brain tissues during henipavirus infection in ferrets. Although the molecular mechanisms governing henipavirus pathogenesis remain unknown, these studies reveal a strategy for dissecting the genetic pathways by which henipavirus infection induces changes in the host response in lung and brain, leading to death.

## Materials and methods

### Ethics statement

Approval for animal experiments was obtained from the Institutional Animal Care and Use Committee, University of Texas Medical Branch (protocol numbers 1102011 and 1106028). Animal work was performed by certified staff in an Association for Assessment and Accreditation of Laboratory Animal Care (AAALAC) approved facility. Animal housing, care and experimental protocols were in accordance with NIH guidelines of the Office of Laboratory Animal Welfare.

### Viruses and cells

The NiV-Malaysia (NiV-M), NiV-Bangladesh (NiV-B) and HeV strains were kindly provided by the Special Pathogens Branch (Centers for Disease Control and Prevention, GA, USA). HeV was isolated from a horse during an outbreak in Brisbane in 1994, NiV-M was isolated from a patient with neurological disease during the 1998 outbreak in Malaysia, and NiV-B was isolated from a patient with severe respiratory distress during the 2004 outbreak in Bangladesh. Low-passage virus stocks were used however the exact passage history was not known. The viruses were propagated on Vero cells (CCL-81; ATCC) in Dulbecco’s modified Eagle’s medium (DMEM) supplemented with L-Glutamine, penicillin, streptomycin and 10% fetal bovine serum (HyClone; Logan UT) at 37°C in humidified CO2 incubator (5%). All infectious work was performed in class II BSC in the biosafety level 4 laboratory (BSL4) at the Galveston National Laboratory, UTMB.

### Animal experiments, monitoring and sample collection

Young female ferrets (5–6 week old from Marshall BioResources, NY) were used in these studies. Prior to transfer to the BSL4 laboratory, temperature transponders (BMDS, Seaford, DE) were implanted subcutaneously in the back of the ferrets for body temperature monitoring and animal identification. Animals were anesthetized by chamber induction (5 liters 100%O_2_/min and 3 to 5% isoflurane) for implantation of temperature transponders, virus challenge and specimen collection.

To determine the minimum dose at which 50% animals succumbed to infection (LD_50_), groups of 4 animals were challenged intranasally (IN) with 10, 10^2^, 10^3^, or 10^5^ tissue culture dose leading to a 50% cytopathic effect (TCID_50_) of NiV-M, NiV-B or HeV in 200μL sterile PBS,100μL for each nare. The LD_50_ was calculated by the Reed-Muench method [54]. Back titration was performed on all challenge stocks, to confirm that the challenge dose was within range.

For direct contact transmission experiments, four ferrets were inoculated IN with 5,000 TCID_50_ of each virus and co-housed with naïve ferrets. Animals were monitored daily for clinical signs, body weight loss and temperature. Nasal washes, oral and rectal swabs were collected from inoculated and naïve ferrets on 1, 3, 5, 7 and 9 dpi. Infected ferrets were euthanized upon signs of severe disease (moribund, labored breathing, paralysis) and naïve ferrets were euthanized 21 dpi.

To compare the pathogenesis of all three viruses, groups of 5 ferrets were infected IN with 5,000 TCID_50_ of NiV-M, NiV-B,or HeV. Control animals received the equivalent volume of sterile PBS. Animals were monitored and evaluated daily for the development and severity of clinical signs and clinical scores were calculated based on behavior, weight loss, respiratory and neurological signs, resulting in a cumulative score as a quantitative assessment of pain (S2 Table). Groups of control ferrets were euthanized on 1 and 5 dpi. Groups of 5 henipavirus infected animals were euthanized on 1, 3, 5 dpi or when animals became moribund, and nasal washes, oral and rectal swabs as well as whole blood samples were collected. Brain (olfactory bulb, frontal, stem, and cerebellum), trachea, left and right lung, liver, spleen, kidney, heart, bladder, urine and skull were sampled for virus isolation, RNA extraction and histology. Following necropsy, lungs were weighed as well as scored for percentage of surface lesions.

Nasal washes, oral and rectal swabs were collected in 1 ml of DMEM supplemented with penicillin and streptomycin and vortexed for 30 sec. Whole blood was collected in EDTA Vacutainers. Following sampling, 140 μl of individual samples was added to 560 μl of AVL viral lysis buffer (Qiagen, Inc) for RNA extraction. For tissue, approximately 100 mg was stored in 1 ml of RNAlater (Qiagen, Inc) to stabilize RNA. RNAlater was completely removed, and tissue was homogenized in 1 ml of Trizol (Sigma).

### Viral RNA isolation and quantitative real-time PCR (RT-PCR) assay

Samples of whole blood, urine, swabs and organ homogenates were inactivated in AVL buffer, and RNA was extracted using the QIAamp viral RNA kit (Qiagen, Inc), according to the manufacturer’s instructions. Real-time quantitative RT-PCR was performed using the QuantiFast Probe RT-PCR kit (Qiagen, Inc), targeting the HeV/NiV P gene. Primers (IDT, San Jose, CA) and dual-labeled 5’FAM/3’TMR probe (TIB MOLBIOL, Adelphia, NJ) sequences used for quantifying the P gene expression level were as follows: P gene NiV/HeV forward, ACATACAACTGGACCCARTGGTT; P gene NiV/HeV reverse, CACCCTCTCTCAGGGCTTGA; P gene probe, 6FAM-ACAGACGTTGTATACCATG—TMR. qRT-PCR components were used at the concentrations recommended by the manufacturer and 5 μl of RNA was added to each reaction and the following thermocycling parameters were used: 50°C for 10min, 95 C for 5 min followed by 40 cycles at 95°C for 10 s, 60°C for 30 s.NiV-M, NiV-B and HeV RNA extracted from the titrated virus stocks were used as a standard curve for each assay, to calculate TCID_50_ equivalents in the samples. Standards and samples were assayed in duplicate using the CFX96 Real Time system combined to a C1000 Thermalcycler (BIO-RAD, Hercules, CA) and data were analyzed with the BIO-RAD CFX manager software (version 2.0).

### Histopathology and immunohistochemistry

All tissues were immersion-fixed in 10% neutral buffered formalin for at least 7 days under BSL4 conditions. Prior to removal from the BSL4 laboratory, formalin was exchanged and specimens were processed under BSL2 conditions the next day. Following fixation, skulls were decalcified using a 20% EDTA solution in sucrose (Newcomer Supply) at room temperature for 3 weeks. The 20% EDTA/sucrose solution was exchanged twice prior to gross sectioning the skull.

Tissues were processed by routine histological methods and sections of tissue (5μm thickness) were stained with Hematoxylin and Eosin (H&E) to examine histological changes. Tissues for immunohistochemistry (IHC) were stained as previously described [8], using a rabbit anti-N-nucleoprotein (N) antibody (kindly provided by Dr. C. Broder, Uniformed Services University, Bethesda, MD).

### Analysis of gene expression profiles by Illumina sequencing

100mg of each tissue was collected in 1ml of Trizol, homogenized and preserved at -70°C until further processing. RNA was isolated according to the manufacturer’s instructions followed by clean-up with Qiagen RNAeasy kit (Qiagen). The resulting RNA samples were analyzed in an Agilent Bioanalyzer 2100 (Agilent, Palo Alto, CA) to ensure that each sample met the quality requirements for sequencing (RIN≥7.5). The cDNA sequencing libraries were prepared using the TruSeq RNA prep kit v2 (Illumina, San Diego, CA). The resulting libraries were quantified by qPCR using the Kappa Illumina quantification kit (Kappa), pooled at equimolar concentrations and sequenced in an Illumina HiSeq2500 (50bp single end). To build the reference library, the ferret transcriptome MusPutFur V1 (NCBI accession number AEYP00000000.1) [16] was filtered so that genes with multiple splicing variants only retained the sequence of their first variant. Bowtie2 was used to align the short-read sequences with respect to the ferret transcriptome [55], and later, the read counts were loaded in MultiExperiment viewer v4.9 [56] and DEGseq with the MA-plot with random sampling model [57] was used to compute the statistical significances. The expression levels with respect to a control group were considered significant when the average normalized read count was ≥10, and DEGseq’s FDR value <0.05, and the normalized fold-change was >±1.5. Each experimental group is composed of 3 animals, except for the brain tissue of the control group (n = 2) and of brain tissue of HeV-Day 5 (n = 2) where one sample from each group was removed from the analysis due to poor data quality. Significantly regulated genes were subjected to functional classification using g:Profiler [58] for Gene Ontology and KEGG Pathways, and Enrichr [59] for protein-protein interaction (PPI) hubs of transcription factors.

The sequencing data was deposited at the NCBI’s Sequence Read Archive under BioProject accession PRJNA289121.

### Host gene expression by Real-time PCR analysis

cDNA was generated using the ImProm II kit (Promega). Real-time PCR was performed in triplicate using primers for the ferret genes CCL2, OAS1, CXCL10, IFNG, CD3E, CD8A and beta-actin, and using Power SYBR Green master mix (Applied Biosystems, Foster City, CA, USA) in an ABI Prism 7900HT instrument (Applied Biosystems). Gene levels were normalized to those from the house-keeping gene beta-actin and statistical analysis was performed using Graph Pad Prism (Version 5.0).

## Supporting information

S1 Fig Changes in bodytemperature during henipavirus infection in ferrets. The average bodytemperature is shown in degrees celcius, per day and per group of ferrets infected with NiV-B, NiV-M or HeV and compared with controls.
(TIF)

S2 Fig Nipah virus antigen in brain of infected ferrets. A representative picture of immunohistochemistry demonstrating NiV-M antigen in brain tissue in infected ferrets.
(TIF)

S3 Fig Henipavirus shedding in ferrets. A comparative study using a dose of 5,000 TCID_50_ for each of the three viruses was performed in ferrets: viral shedding in the ferret secretions was measured by quantitative RT-PCR in nasal washes (A), oral swabs (B), rectal swabs (C) and urine (D). Samples were harvested from 6-week old ferrets infected with NiV-B (grey), NiV-M (black solid) and HeV (black dashed) on various days post infection as described in Materials and Methods. Samples from 5 animals per group were analyzed at each time point. * p<0.01, two-way ANOVA, Bonferroni’s multiple comparison test. Error bars represent standard deviations.
(TIF)

S4 Fig Host gene expression in lung and brain of henipavirus infected ferrets. Relative mRNA expression of CXCL10, OAS1, IFNg, CD3E and CD8A in lung and brain tissue at various days post infection.
(TIF)

S1 Table Virus shedding and seroconversion in direct contact study. (DOCX)

S2 Table Quantitative assessment of pain independent variable score. (DOCX)
