# Supplementary figures and images for: Host gene expression profiles in ferrets infected with genetically distinct henipavirus strains
Source: PLoS Negl Trop Dis. 2018 Mar 14;12(3):e0006343. doi: 10.1371/journal.pntd.0006343 (PMC5868854; doi:10.1371/journal.pntd.0006343)

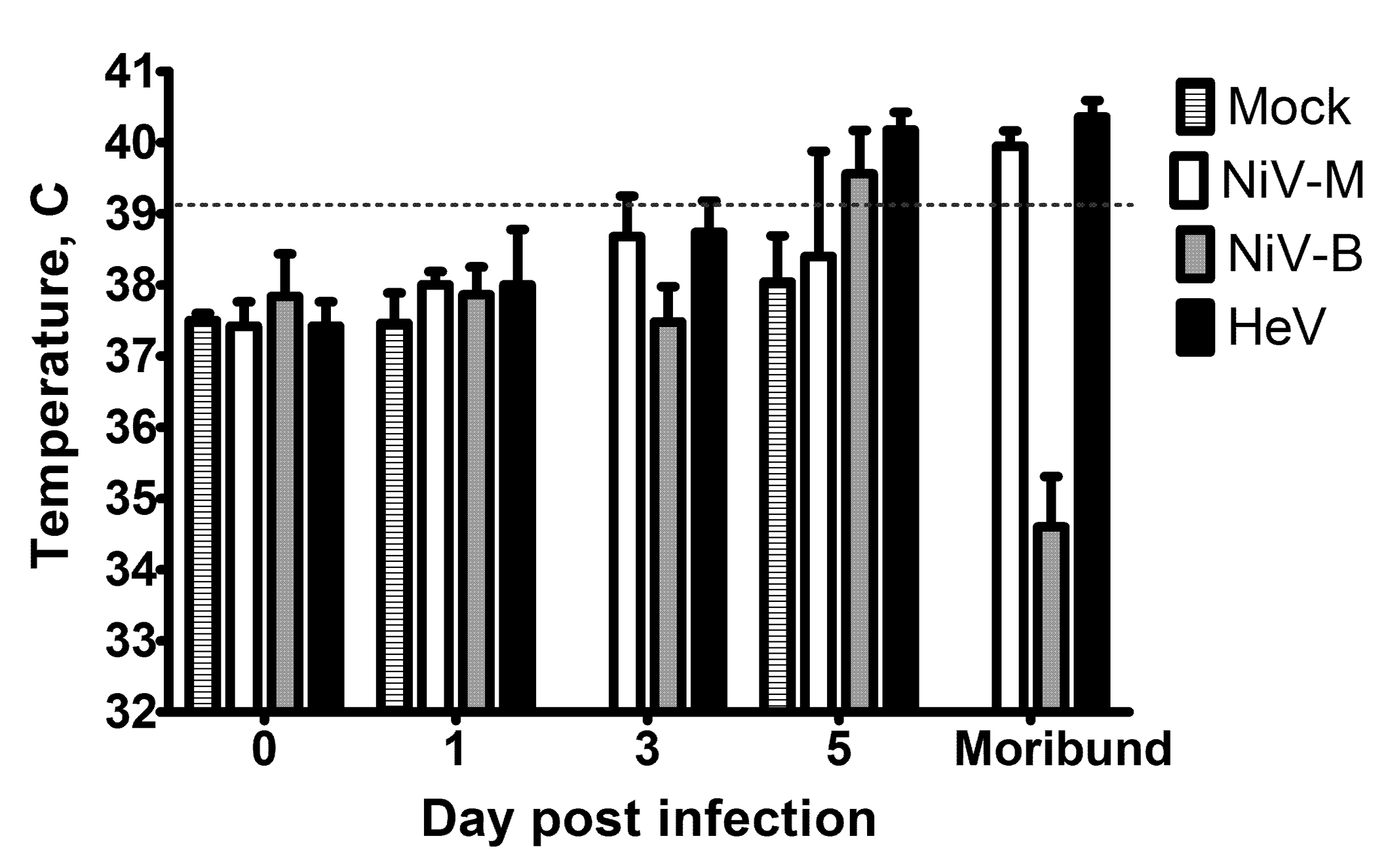

Supplement: S1 Fig — The average bodytemperature is shown in degrees celcius, per day and per group of ferrets infected with NiV-B, NiV-M or HeV and compared with controls. (TIF) [file pntd.0006343.s001.tif]

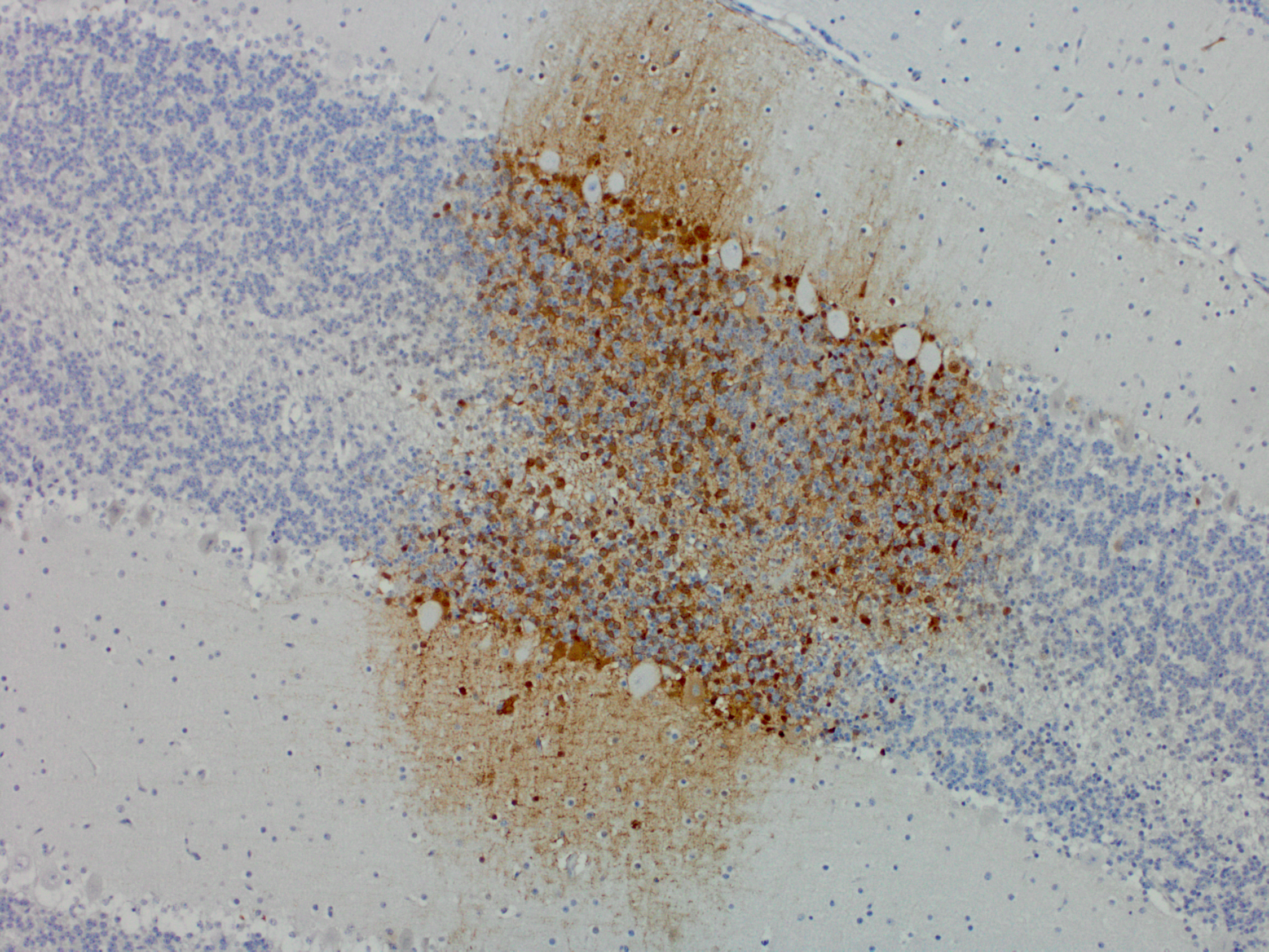

Supplement: S2 Fig — A representative picture of immunohistochemistry demonstrating NiV-M antigen in brain tissue in infected ferrets. (TIF) [file pntd.0006343.s002.tif]

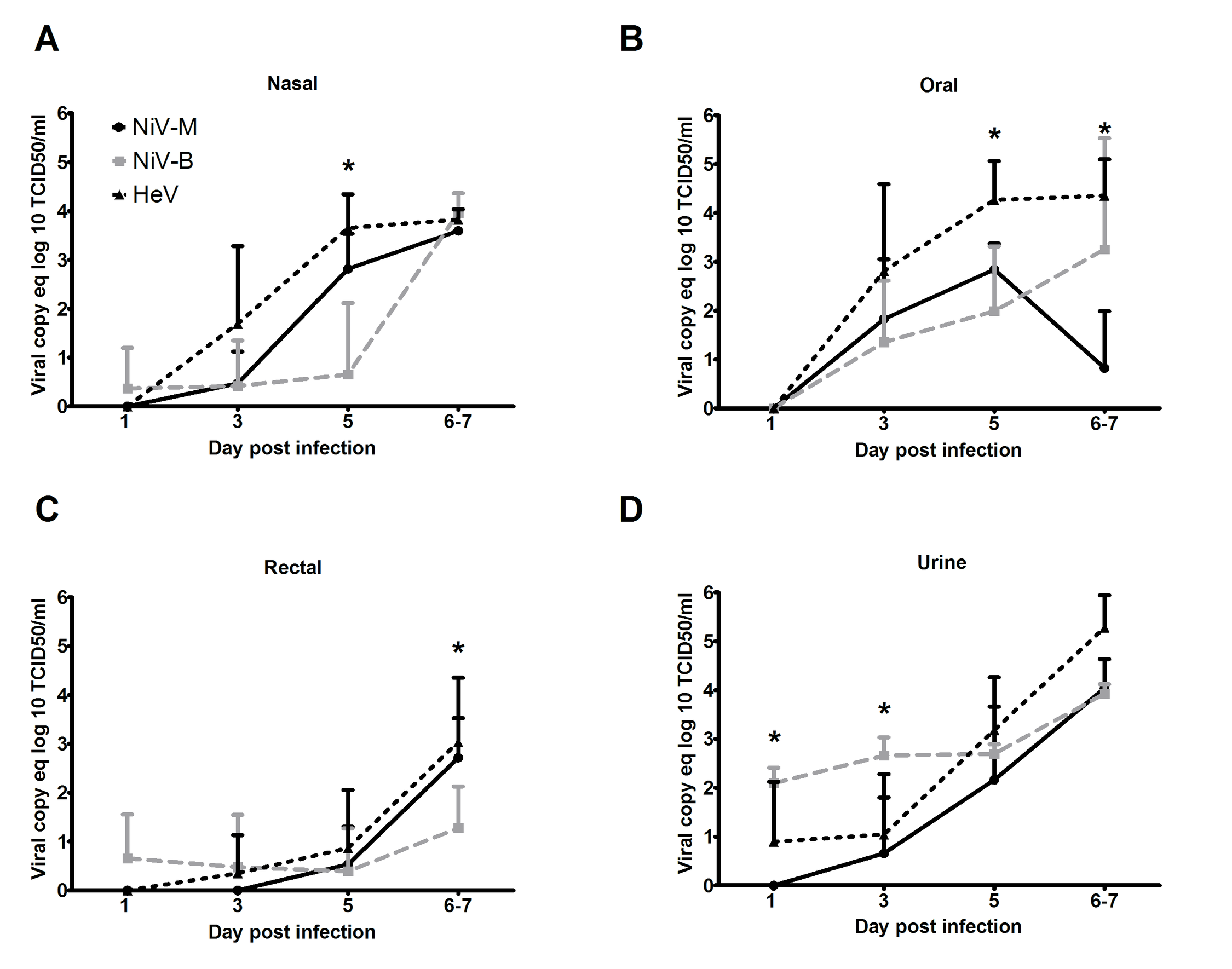

Supplement: S3 Fig — A comparative study using a dose of 5,000 TCID50 for each of the three viruses was performed in ferrets: viral shedding in the ferret secretions was measured by quantitative RT-PCR in nasal washes (A), oral swabs (B), rectal swabs (C) and urine (D). Samples were harvested from 6-week old ferrets infected with NiV-B (grey), NiV-M (black solid) and HeV (black dashed) on various days post infection as described in Materials and Methods. Samples from 5 animals per group were analyzed at each time point. * p<0.01, two-way ANOVA, Bonferroni’s multiple comparison test. Error bars represent standard deviations. (TIF) [file pntd.0006343.s003.tif]

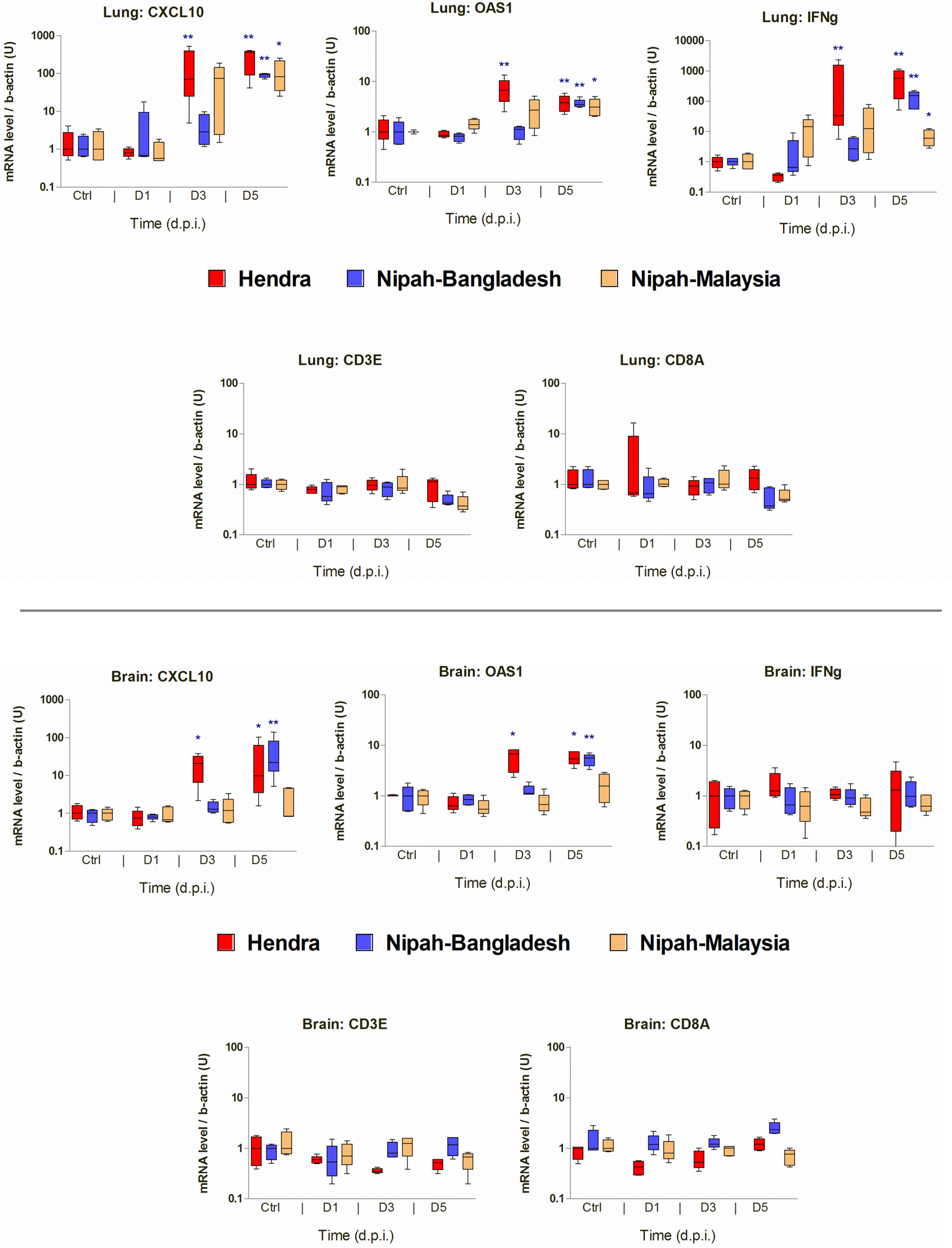

Supplement: S4 Fig — Relative mRNA expression of CXCL10, OAS1, IFNg, CD3E and CD8A in lung and brain tissue at various days post infection. (TIF) [file pntd.0006343.s004.tif]
