# Supplementary material for: Host gene expression profiles in ferrets infected with genetically distinct henipavirus strains
Source: PLoS Negl Trop Dis. 2018 Mar 14;12(3):e0006343. doi: 10.1371/journal.pntd.0006343 (PMC5868854; doi:10.1371/journal.pntd.0006343)
Supplement: S1 Table — (DOCX) [file pntd.0006343.s005.docx]

**S1 Table. Virus shedding and seroconversion in direct contact study**

|  |  | Infected | Contact |
| --- | --- | --- | --- |
| Virus shedding | Nasal wash | pos | neg |
|  | Oral swab | pos | neg |
|  | Rectal swab | pos | neg |
| Serology | ELISA^1^ | pos | neg |
|  | VNT^2^ | pos | neg |

^1^ ELISA = Enzyme-Linked Immuno Sorbent Assay; ^2^ VNT = Virus neutralization test; pos = positive; neg = negtaive
