# Supplementary material for: Host gene expression profiles in ferrets infected with genetically distinct henipavirus strains
Source: PLoS Negl Trop Dis. 2018 Mar 14;12(3):e0006343. doi: 10.1371/journal.pntd.0006343 (PMC5868854; doi:10.1371/journal.pntd.0006343)
Supplement: S2 Table — (DOCX) [file pntd.0006343.s006.docx]

**S2 table. Quantitative Assessment of Pain Independent Variable Score**

Neurological Signs:

0 Normal

1 Mild paraparesis

2 Moderate paraparesis

3 Severe quadriparesis/quadriparalysis/seizures

4 Moribund

Respiratory Signs:

0 Normal

1 Small change of potential significance

2 Altered breathing

3 Labored breathing

Behavior:

0 Normal

1 Lack of grooming

2 Abnormal or hunched posture

3 Less mobile, less alert, inactive when activity expected

Weight loss:

0 Normal

1 < 10%

2 10-15%

3 15-20%

4 >20%
